# Don't hide the instruction manual: A dynamic trade-off between using internal and external templates during visual search

**DOI:** 10.1167/jov.23.7.14

**Published:** 2023-07-24

**Authors:** Alex J. Hoogerbrugge, Christoph Strauch, Tanja C. W. Nijboer, Stefan Van der Stigchel

**Affiliations:** 1 Experimental Psychology, Helmholtz Institute, Utrecht University, The Netherlands; 2 Center of Excellence for Rehabilitation Medicine, UMC Utrecht Brain Center, University Medical Center Utrecht, The Netherlands; 3 De Hoogstraat Rehabilitation, Department of Rehabilitation, Physical Therapy Science & Sports, UMC Utrecht Brain Center, University Medical Center Utrecht, The Netherlands

**Keywords:** visual search, visual working memory, search template, resampling, trade-off, multi-template search

## Abstract

Visual search is typically studied by requiring participants to memorize a template initially, for which they subsequently search in a crowded display. Search in daily life, however, often involves templates that remain accessible externally, and may therefore be (re)attended for just-in-time encoding or to refresh internal template representations. Here, we show that participants indeed use external templates during search when given the chance. This behavior was observed during both simple and complex search, scaled with task difficulty, and was associated with improved performance. Furthermore, we show that participants used external sampling not only to offload memory, but also as a means of verifying whether the template was remembered correctly at the end of trials. We conclude that the external world may not only provide the challenge (e.g., distractors), but may dynamically ease search. These results argue for extensions of state-of-the-art models of search, because external sampling seems to be used frequently, in at least two ways and is actually beneficial for task performance. Our findings support a model of visual working memory that emphasizes a resource-efficient trade-off between storing and (re)attending external information.

## Introduction

When we shop for groceries, lay a jigsaw puzzle, or attempt to assemble a piece of Swedish furniture, we must perform visual search. To find an object (e.g., a specific screw), we must actively keep a search template in working memory, and then search for it amongst other items. Once we are confident that an attended stimulus matches our template, search is finalized (Palmer, Verghese, & Pavel, 2000; Olivers & Eimer, 2011; Wolfe, 2021).

It is no surprise that visual search has been well-investigated for almost a century, given how fundamental this process is for everyday life (e.g., Wolfe, 2010, 2021; Titchener, 1924; Kingsley, 1932). In traditional paradigms, a template has to be maintained in visual working memory (VWM) throughout search, after transient and singular presentation (e.g., Wolfe, 2021). After the offset of the template, participants are presented with a search array and have to indicate whether the target was present. Exhaustive and well-established models for this visual search (e.g., Wolfe, 1994, 2021) explain not only the underlying processes, but also when and why search goes wrong. For instance, the difficulty of visual search scales with stimulus complexity, set size, and many other factors (Anderson, 1996; Wolfe, 1998; Cain, Adamo, & Mitroff, 2013; Hulleman & Olivers, 2017; Wolfe, 2021).

Although the conventional experimental set-up has provided many insights into search, many instances of search in daily life differ. Think of your personal experience when it comes to assembling a piece of Swedish furniture, for instance: When searching for two unique screws from a bag full of differing types of screws, we may regularly fail to identify both of our targets in the first attempt. Luckily, we can always choose to memorize and search for one screw first, refer back to the instruction manual, and then search for the other. Similarly, we can look back at the instruction manual to refresh our template representations in VWM whenever we feel insufficiently confident that we indeed found the screw that we were looking for.

The external world can thus often help us to refresh the template throughout search, effectively decreasing the burden on VWM. Then, the external world may not only provide the challenge (e.g., the search display), but also ease the challenge, by allowing to resample the template. Indeed, during many tasks, humans look back and forth at instructions in order to help them succeed (Hayhoe, Shrivastava, Mruczek, & Pelz, 2003; Droll & Hayhoe, 2007; Hansen, Mardanbegi, Biermann, & Bækgaard, 2018; Alfandari, Belopolsky, & Olivers, 2019; Sullivan, Ludwig, Damen, Mayol-Cuevas, & Gilchrist, 2021). This behavior is in line with earlier findings on mental effort and memory, which indicate that offloading memory is preferred as much as possible over storing internally, by (re)sampling from the environment in a just-in-time manner (O’Regan, 1992; Hayhoe et al., 2003; Triesch, Ballard, Hayhoe, & Sullivan, 2003; Droll, Hayhoe, Triesch, & Sullivan, 2005; Risko & Dunn, 2015; Risko & Gilbert, 2016; Melnik, Schüler, Rothkopf, & König, 2018; Somai, Schut, & Van der Stigchel, 2020; Van der Stigchel, 2020; Draschkow, Kallmayer, & Nobre, 2021).

It is currently unknown how observers balance between internal storage of the template and sampling of the external world in search. We therefore asked whether—and to which degree—participants make use of the option to resample not only the search array, but also the template, when given the chance. To answer these questions, we adopted the following reasoning:

• If participants resample templates throughout search when given the chance, this would indicate that they use template availability as a means of relying on the external world relative to relying on VWM.
• When templates remain available, the amount of resampling indicates the degree of reliance on the external world as compared to VWM. This reliance may also change as a result of task difficulty.
• If the amount of resampling is positively associated with better accuracy or completion times, this would indicate a quantifiable benefit of external sampling on search.

To investigate these questions, participants performed visual search tasks with single- and multi-template search, as well as conditions in which the template(s) remained available throughout a trial, or needed to be encoded up front.

## Experiment 1

### Methods

All data together with analysis scripts and supplementary materials may be retrieved via the Open Science Framework https://osf.io/ec7b6/.

#### Participants and procedure

Nineteen participants performed the experiment, of which two were excluded from analysis due to technical issues and one dropped out during data collection. Thus sixteen participants (8 female, 8 male, age 18–29) were included in the analyses.

Before the task, participants read the information letter, signed an informed consent form, and indicated their age and gender. Participants received €7 per hour or course credits, with Experiment 1 taking approximately 60 minutes. The experiment was preceded by four practice trials. The study was approved by the faculty ethics board of Utrecht University, adhering to the declaration of Helsinki.

#### Apparatus

Monocular gaze location was recorded with an EyeLink 1000+, at a sampling rate of 1 kHz. Stimuli were presented on a 27” 2560 × 1440 LCD monitor with a refresh rate of 100 Hz. Participants were seated and stabilized with a chin- and forehead rest at 67.5 cm from the monitor. The experiment was implemented using PyGaze (Dalmaijer, Mathôt, & Van der Stigchel, 2014).

All gaze metrics are reported in degrees of visual angle (°). Before the start of the experiment, and between each block, the eye tracker was calibrated and validated with a nine-dot grid, allowing a mean error of 0.5° and a maximum per-dot error of 1.0°. The quality of calibration was automatically evaluated throughout the experiment while each pretrial fixation cross was presented. If the calibration error exceeded 1.5° over more than two trials, the eye tracker was recalibrated.

#### Task and design

Participants performed a visual search task, in which the screen was divided into two sections; a template area and a search area, divided by a vertical line. The template area occupied the leftmost quarter (12.7°) of the screen and contained one or four templates, dependent on condition. The search area occupied the rightmost three-quarters (38.1°) of the screen and contained either one target (matching exactly one of the templates) and 10 distractors in target-present trials, or 11 distractors in target-absent trials. Distractors were randomly picked and could therefore be presented multiple times within the search array. Memory loads of one and four templates were chosen such that there were conditions with the minimum required VWM load for any given search task (one template), and conditions that required VWM to be loaded to (or above) capacity if all templates were encoded at once (four templates; Vogel & Awh, 2008; Luck & Vogel, 2013; Adam, Vogel, & Awh, 2017). Seventy-five percent of trials were target-present trials. Stimuli were spread out such that participants could not fixate templates and search items simultaneously.

**Figure 1.**
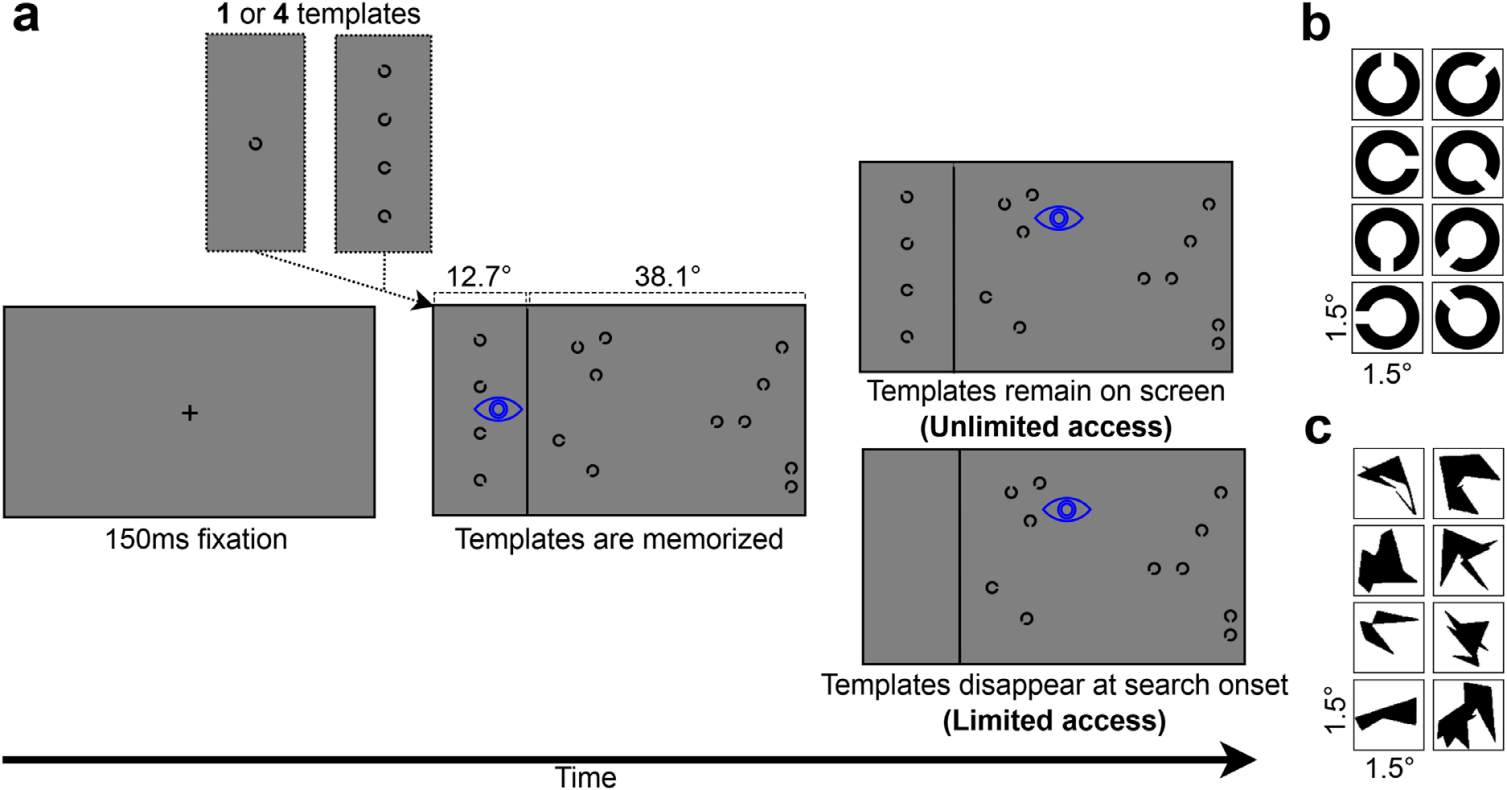
(a) Sequence of a trial. Trials could contain either one template or four templates. In the unlimited access conditions, templates would remain on the screen throughout a trial. In the limited access conditions, templates would disappear as soon as search started. The vertical line was always present throughout a trial. Stimulus size is not to scale. (b) The eight Landolt C’s used in Experiment 1. (c) The eight original stimuli used in Experiment 2 (Arnoult, 1956). Each stimulus could be shown in one of four rotations, thus creating 32 stimuli. All stimuli occupied approximately 1.5° of visual angle.

The stimulus set consisted of Landolt C’s in eight possible orientations, commonly used in visual search tasks (e.g., Becker, 2011; Carlisle, Arita, Pardo, & Woodman, 2011; Smith, Crabb, & Garway-Heath, 2011; Vanyukov, Warren, Wheeler, & Reichle, 2012; Alfandari et al., 2019; Palmer, Van Wert, Horowitz, & Wolfe, 2019). Each stimulus was approximately 1.5° in size (Figure 1b).

Before the start of each trial, a central fixation cross was shown, and the trial would only start if a fixation was detected at that location. Participants memorized the template(s) in the template area, and searched for them in the search area indicating for each trial whether one of the stimuli in the search area matched a template (by pressing the “z”-key) or not (“/”-key). After each trial, they received feedback, with the screen showing either “Correct” or “Incorrect” in blue or red text, respectively (Figure 1a). Trials were marked as invalid if the participant indicated that gaze contingent template disappearance did not work as intended.

In the unlimited access condition, templates were visible throughout each entire trial. This allowed participants to gaze back at the templates (resample). The limited access condition followed a classical visual search paradigm by requiring participants to memorize as many templates as possible at once; when participants’ gaze crossed the dividing line from the template area toward the search area for the first time, the templates were removed from the screen and could not be sampled again for the remainder of the trial (Figure 1a).

The task thus contained four conditions: 1) one template with unlimited access, 2) one template with limited access, 3) four templates with unlimited access or 4) four templates with limited access. These conditions are referred to as 1-Unlimited, 1-Limited, 4-Unlimited, and 4-Limited.

Participants performed 60 trials in each of these four conditions; the sequence of conditions was counterbalanced following a Latin square design.

#### Analysis

We report three outcome variables. 1) Gaze Crossings to Template was extracted by counting the number of saccades which started in the search area and landed in the template area. This variable is representative of the amount of (re)sampling. Because each trial started with a central fixation cross, the minimum number of crossings was always 1, and any value above is indicative of resampling. 2) Balanced Accuracy was computed by calculating recall scores (hits divided by number of target-present trials and correct rejections divided by number of target-absent trials, respectively), and taking a weighted average of the two—thereby taking into account the unequal proportion of target-present and -absent trials (implemented using balanced_accuracy_score in scikit-learn; Pedregosa et al., 2011). Balanced Accuracy ranges from 0 to 1, with 0.5 denoting chance-level performance (Brodersen, Ong, Stephan, & Buhmann, 2010). 3) Completion Time was computed as the time in seconds from the first frame in which the trial screen was visible until a keypress was recorded.

For all three outcome variables, trials that were marked as invalid were discarded from the analysis. For Gaze Crossings to Template and completion time, only target-present trials and trials with a correct response were considered. Additionally, trials with values beyond the overall 99th percentile were removed. Outcomes of statistical tests with and without these corrections did not substantially differ. In total, 1.5% of trials were marked as invalid, and 1.7% of trials were discarded as outliers.

The median (*Mdn*) and median absolute deviation (*MAD*) are reported for group-level outcomes instead of the mean and standard deviation, to better account for non-normally distributed data and group-level comparisons.

Analyses were performed in JASP v0.16.3 (JASP Team, 2022, default priors were used for Bayesian statistics). We report outcomes of Bayesian analyses of variance and *t* tests, and indicate whether those tests were performed directionally (*BF*_+0_, *BF*_−0_) or nondirectionally (*BF*_10_). Effect sizes (Cohen’s *d* and $\eta_{p}^{2}$, obtained from classical parametric tests) are reported alongside Bayes factors. If the assumption of normality was violated, a Bayesian Wilcoxon signed rank test is reported instead, although parametric and nonparametric tests conceptually provided very similar outcomes.

An overview of statistical outcomes is reported in the Supplementary Materials.

### Results

**Figure 2.**
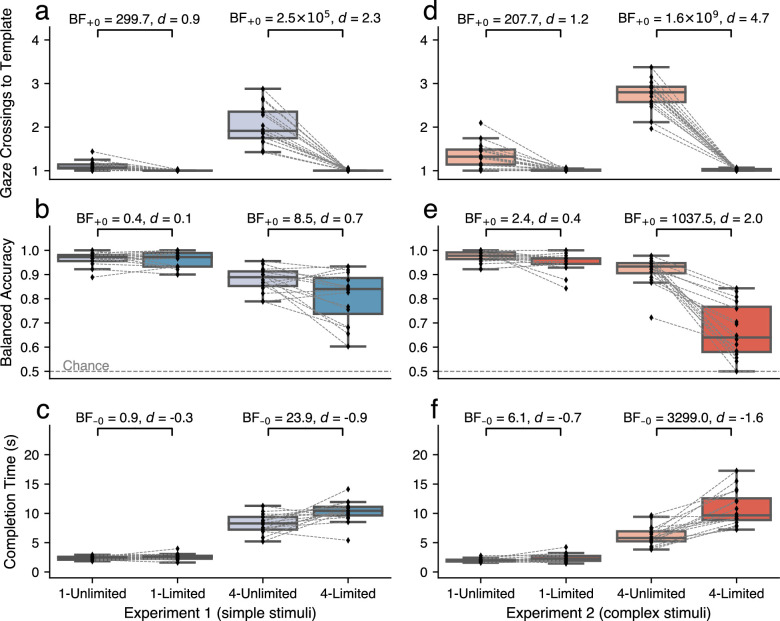
Outcome measures of Experiment 1 (left) and Experiment 2 (right). (a, d) The number of times the gaze crossed from the search area to the template area, as a measure of (re)sampling. Because each trial started with a central fixation cross, the minimum number of crossings was always 1, and any value above is indicative of resampling. (b, e) Balanced Accuracy, which takes into account an unequal proportion of target-present and -absent trials. Chance performance = 0.5. (c, f) Trial completion time in seconds, measured from trial start until keypress. Note: All panels except b and e visualize data of correctly answered and target-present trials only. Diamond markers denote individual participants.

Participants would regularly resample if given the chance, even sometimes when only one template needed to be memorized (main effect of template availability *BF*_10_ = 6706.83, $\eta_{p}^{2}=0.89$; Figure 2a). When searching for a single template, participants made slightly more than one gaze crossing per trial from the search area to the template area if templates remained available throughout the trial (1-Unlimited; *Mdn* = 1.07, *MAD* = 0.08; 2.6% of trials contained a second crossing). If the template could only be sampled once, participants did not make additional gaze crossings back to the template area (1-Limited; *Mdn* = 1.0, *MAD* = 0.0; *BF*_+0_ = 299.7, *d* = 0.9). This pattern was more accentuated when participants had to memorize four items (main effect number of templates *BF*_10_ = 497.13, $\eta_{p}^{2}=0.80$; interaction effect *BF*_10_ = 5.30 × 10^10^, $\eta_{p}^{2}=0.79$). Here, participants made more crossings when templates remained available (4-Unlimited; *Mdn* = 1.91, *MAD* = 0.46) than when access to the templates was limited, where they made only the initial crossing (4-Limited; *Mdn* = 1.0, *MAD* = 0.0; *BF*_+0_ = 2.5 × 10^5^, *d* = 2.3).

Overall, there was a main effect of the number of templates on Balanced Accuracy (*BF*_10_ = 8358.19, $\eta_{p}^{2}=0.79$; Figure 2b), but not of template availability (*BF*_10_ = 2.03, $\eta_{p}^{2}=0.32$). The accuracy was equal between the 1-Unlimited condition (*Mdn* = 0.97, *MAD* = 0.03) and the 1-Limited condition (*Mdn* = 0.97, *MAD* = 0.04; *BF*_+0_ = 0.4, *d* = 0.1), suggesting that resampling had no immediate benefit on accuracy in single-template search. With four templates, however, a benefit of unlimited access to the templates was observed, with higher accuracy in the 4-Unlimited condition (*Mdn* = 0.89, *MAD* = 0.05) than in the 4-Limited condition (*Mdn* = 0.84, *MAD* = 0.11; *BF*_+0_ = 8.5, *d* = 0.7; interaction effect *BF*_10_ = 10.26, $\eta_{p}^{2}=0.33$). These findings highlight that the number of templates and template availability dynamically affected accuracy on the task.

A main effect of template availability highlights an overall benefit of the possibility to resample on completion time (*BF*_10_ = 7.33, $\eta_{p}^{2}=0.43$; Figure 2c), although this effect was driven by differences in four-template search and not in single-template search (interaction effect *BF*_10_ = 91.89, $\eta_{p}^{2}=0.42$). Specifically, template availability did not benefit speed when participants memorized one template. Completion times were similar in the 1-Unlimited condition (*Mdn* = 2.49 s, *MAD* = 0.33) and 1-Limited condition (*Mdn* = 2.60 s, *MAD* = 0.43; *BF*_−0_ = 0.9, *d* = −0.3). In the 4-Unlimited condition (*Mdn* = 8.28 s, *MAD* = 1.69), participants were two seconds faster than in the 4-Limited condition (*Mdn* = 10.44 s, *MAD* = 1.11; *BF*_−0_ = 23.9, *d* = −0.9). As such, template availability decreased search completion time, but only when searching for multiple templates.

### Interim discussion

In Experiment 1, we investigated whether participants preferred to rely on the external world rather than taxing VWM—and if so, what the extent of this reliance was and whether it changed as a result of task difficulty. Last, we investigated whether there was a quantifiable benefit of this reliance on behavior.

Participants regularly resampled the template area when templates remained available throughout the trial, sometimes even when only one simple template needed to be memorized. Furthermore, this effect was greater in multi-template search compared to single-template search. This indicates that participants often relied on availability of templates when possible, but that the degree of this reliance was dependent on task difficulty.

When memorizing one template, the ability to resample was not linked to an observable benefit on classical behavioral outcomes such as accuracy or completion time. However, a benefit of the ability to resample did emerge with four templates instead of one, which indicates that the usefulness of being able to resample becomes more pronounced when the demand on VWM is increased.

Because participants performed very well with one template—which suggests possible floor/ceiling effects—and because the stimuli relied on just one feature (opening direction), we sought to extend the observed phenomena to more complex visual stimuli in Experiment 2.

For Experiment 2 we, therefore, posited:

• If search difficulty was indeed to affect the degree of reliance on the external world as opposed to VWM, then similar effects as in Experiment 1 should be observed, but more pronounced in nature with complex stimuli. This should be observable in single-template search, and be further accentuated in multi-template search.

## Experiment 2

### Methods

Experiment 2 followed the same design and procedure as Experiment 1, but with different stimuli.

#### Participants

Eighteen participants performed the experiment, of which two were excluded due to technical issues. Of the remaining 16 participants (7 female, 9 male, age 18–29 years), seven had also participated in Experiment 1. Experiment 2 took approximately 90 minutes to complete.

#### Stimuli

Stimuli (Figure 1c) were a subset of complex shapes introduced by Arnoult (1956), which have been previously employed in VWM research (e.g., Somai et al., 2020; Sahakian, Gayet, Paffen, & Van der Stigchel, 2023).

To determine which of the original 30 stimuli were most difficult to verbalize, an online pilot study was run (*N* = 48). Participants indicated which word or name they would assign to each of the stimuli. We then computed the consensus (the percentage of identical or semantically similar responses) and selected the eight stimuli for which consensus was lowest (*M* consensus of used stimuli = 43%; *M* of all stimuli = 61%).

Each of the eight selected stimuli could be shown in 4 configurations (90° rotations), resulting in 32 stimuli. Template and target were considered to be matched only if both the shape and rotation were identical.

### Results

Replicating Experiment 1, participants resampled more frequently when templates remained externally available (main effect *BF*_10_ = 3.69 × 10^5^, $\eta_{p}^{2}=0.92$; Figure 2d), and this was again stronger in four-template search than in single-template search (interaction effect *BF*_10_ = 6.68 × 10^21^, $\eta_{p}^{2}=0.97$). Participants made a greater number of crossings from the search area to the template area in the 1-Unlimited condition (*Mdn* = 1.32, *MAD* = 0.27; 8.7% of trials contained a second crossing) as compared with the 1-Limited condition (*Mdn* = 1.0, *MAD* = 0.0; *BF*_+0_ = 207.7.6, *d* = 1.2). They also made a greater number of crossings in the 4-Unlimited condition (*Mdn* = 2.8, *MAD* = 0.26) as compared with the 4-Limited condition (*Mdn* = 1.03, *MAD* = 0.05; *BF*_+0_ = 1.6 × 10^9^, *d* = 4.7). Beyond replication of Experiment 1, these accentuated effects indicate that the introduction of more complex search templates indeed led to more external sampling.

Overall, unlimited template availability had a positive effect on accuracy (*BF*_10_ = 1323.97, $\eta_{p}^{2}=0.81$; Figure 2e). This effect was not previously present, showing that the introduction of complex stimuli indeed affected task performance. This was again dynamically altered by the number of templates (interaction effect *BF*_10_ = 2.13 × 10^8^, $\eta_{p}^{2}=0.77$). The Balanced Accuracy was approximately equal between the 1-Unlimited condition (*Mdn* = 0.98, *MAD* = 0.03) and the 1-Limited condition (*Mdn* = 0.97, *MAD* = 0.03; *BF*_+0_ = 2.4, *d* = 0.4). Showing a much more pronounced effect than in Experiment 1, however, participants performed the task substantially more accurately in the 4-Unlimited condition (*Mdn* = 0.93, *MAD* = 0.03) than in the 4-Limited condition, where some participants even performed near chance level (*Mdn* = 0.64, *MAD* = 0.11; *BF*_+0_ = 1037.5, *d* = 2.0). These findings again highlight that template availability can benefit accuracy on the task, but more substantially so with complex stimuli than with simple stimuli.

Participants were consistently faster when they could resample (main effect *BF*_10_ = 180.74, $\eta_{p}^{2}=0.72$; Figure 2f), but this benefit was greater in four-template search than in single-template search (interaction effect *BF*_10_ = 2.95 × 10^5^, $\eta_{p}^{2}=0.73$). Participants were slightly faster in the 1-Unlimited condition (*Mdn* = 1.96 s, *MAD* = 0.27) than in the 1-Limited condition (*Mdn* = 2.29 s, *MAD* = 0.66; *BF*_−0_ = 6.1, *d* = −0.7), which indicates a small benefit of the ability to resample on task completion time. The benefit of template availability on completion times was also observed in the 4-Unlimited condition (*Mdn* = 5.79 s, *MAD* = 01.45), showing almost a halving of the completion time as compared with the 4-Limited condition (*Mdn* = 9.67 s, *MAD* = 2.71; *BF*_−0_ = 3299.0, *d* = −1.6). Overall, these findings show again that the benefit of template availability on completion time was more pronounced in four- versus single-template search, and in complex- versus simple templates.

### Interim discussion

In both experiments, participants made use of the possibility to resample templates, primarily when four items needed to be memorized. The possibility to resample templates was associated with shorter completion times and higher accuracy.

But why did template availability benefit completion times, given that this would require more large saccades back and forth between the template and search areas? And to what end did participants resample? Was it to encode subsets of templates after each gaze crossing or was it to refresh (double-check) existing representations in VWM? Last, one may ask whether double-checking was actually beneficial for search accuracy. We address these questions in the following section.

## How was template availability used?

### Just-in-time sampling was linked to shorter completion times than fully loading VWM

#### Analysis

We report two outcome variables aimed at uncovering why participants were slower at the task when they could not resample. These variables inform us how much time was spent encoding templates (Irwin, 2004; Koevoet, Naber, Strauch, Somai, & Van der Stigchel, 2023). 1) Total Sampling Duration in seconds provides the overall dwell time in the template area, and was computed as the summed duration of all fixations in the template area within each trial. 2) Template Fixation Duration in milliseconds arguably indicates how elaborately participants encoded templates, and was extracted by computing the median duration of all fixations in the template area within each trial.

The outcome variables were aggregated by the median per participant, per condition.

#### Results

**Figure 3.**
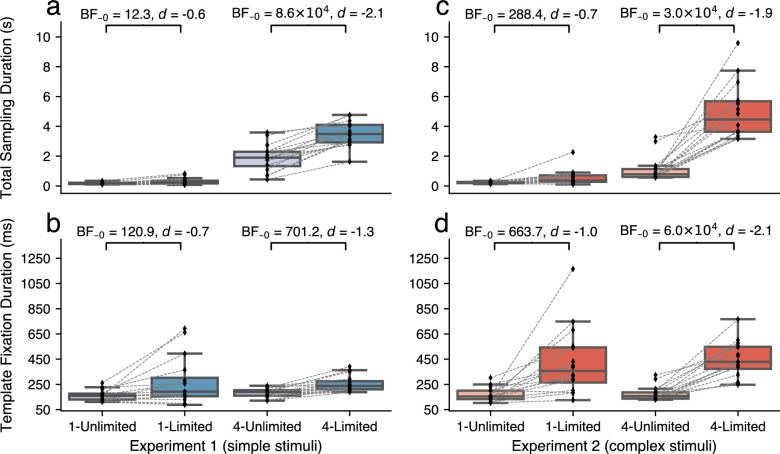
Increased dwell times and fixation durations when participants could not resample. (a, c) Time spent sampling the template area (in seconds; sum of fixation durations). (b, d) Median duration of fixations in the template area (in milliseconds) as a measure of the attempted depth of encoding of individual templates. Note: All panels visualize data of correctly answered and matching trials only. Diamond markers denote individual participants.

Participants spent more time fixating the template area when they could not resample (Figures 3a, c). There were main effects of template availability in both experiments (Experiment 1 *BF*_10_ = 362.45, $\eta_{p}^{2}=0.66$; Experiment 2 *BF*_10_ = 925.9, $\eta_{p}^{2}=0.77$), and this effect was stronger when four templates needed to be encoded (interaction effects Experiment 1 *BF*_10_ = 4.74 × 10^6^, $\eta_{p}^{2}=0.85$; Experiment 2 *BF*_10_ = 1.12 × 10^9^, $\eta_{p}^{2}=0.82$).

There was no main effect of template availability on total search duration in either experiment (*BF*_10_ = 0.26, $\eta_{p}^{2}=0.001$; see Supplementary Materials Figure 1), meaning that increased template sampling duration was the main cause of the increased trial completion times when templates could not be resampled.

Furthermore, participants fixated longer on individual templates when those templates could not be resampled, regardless of the number of templates that needed to be memorized (main effects of template availability Experiment 1 *BF*_10_ = 26.0, $\eta_{p}^{2}=0.54$; Experiment 2 *BF*_10_ = 2419.8, $\eta_{p}^{2}=0.74$; Figures 3b, d), which suggests that participants attempted to encode templates more deeply when they knew that they could not resample later.

Together, these findings show that participants spent more time encoding templates when they could not resample them later, which was linked to longer completion times. When templates could be resampled, it, therefore, seems that encoding fewer templates, and encoding them less deeply, was a relatively efficient strategy which compensated for the added time cost of making multiple gaze crossings.

### Templates were encoded just in time or refreshed

#### Analysis

Data of Experiments 1 and 2 were combined (*N* = 32), using all trials from the conditions in which participants could resample (1-Unlimited and 4-Unlimited, including target-absent and incorrect trials). Outcomes were conceptually similar when including only target-present and correct trials.

We explored with two outcome variables whether resampling was used to just-in-time encode subsets of templates, or whether it was used to refresh existing representations in VWM: 1) Onset of each gaze crossing to the template area, expressed as a percentage of trial duration. Onsets were defined as the onset of saccades which left the search area and landed in the template area. 2) The number of unique templates fixated after each crossing. By definition, this value was always 1 in the 1-Unlimited condition, because only one template was present. In the 4-Unlimited condition, this value could range from 1 to 4.

We next calculated these outcome variables based on whether they described the first, second, third, or fourth crossing within a trial. Too few third and fifth crossings were made in the 1-Unlimited and 4-Unlimited conditions respectively, so those crossings and subsequent crossings are not reported. Crossings in which no templates were fixated were excluded (4.3%). Four out of 32 participants did not make any second crossings in the 1-Unlimited conditions and were, therefore, excluded, leaving 28 remaining participants for analysis of the 1-Unlimited condition. Additionally, one participant did not make any third or fourth crossings in the 4-Unlimited conditions, and was therefore excluded, leaving 31 remaining participants for analysis of the 4-Unlimited condition. Per outcome variable, values beyond the overall 99th percentile were excluded as outliers. The outcome variables were then aggregated by the mean per participant, per condition.

#### Results

**Figure 4.**
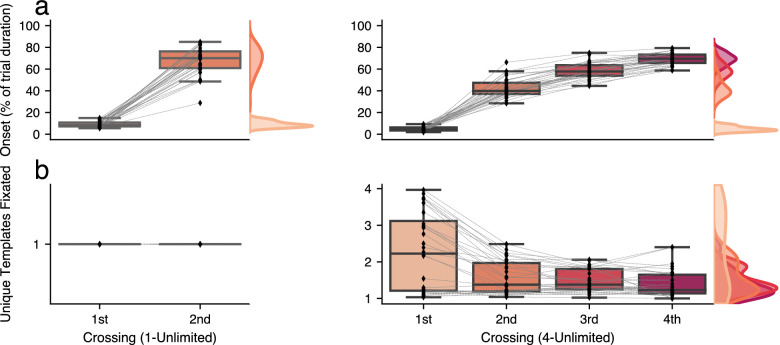
Resampling could be used to refresh the template representation in VWM or to encode templates just-in-time. (a) The onset of crossings toward the template area, expressed as a percentage of trial duration. (b) The number of unique templates that were fixated per crossing. In the 1-Unlimited condition, only one unique template could be fixated. Note: Outcome measures of Experiments 1 and 2 combined. Diamond markers denote individual participants. Not all participants made multiple crossings in all trials.

In both conditions, participants made their first crossing almost immediately after trial onset (1-Unlimited *Mdn* =8.4%, *MAD* =2.64%; 4-Unlimited *Mdn* =4.2%, *MAD* =2.08%), and thus did not elaborately inspect the search array before crossing towards the template area (Figure 4a). When participants needed to memorize one template, secondary gaze crossings were made relatively late in the trial (*Mdn* =70.0%, *MAD* =10.65%), which suggests that this crossing often served to double-check whether the target was indeed found (or verifying that it was absent), by refreshing the template representation in VWM.

When four templates needed to be memorized, secondary crossings were made relatively earlier in the trial (*Mdn* =39.9%, *MAD* =6.63%) than in the one-template condition (*BF*_10_ = 4437.2, *d* = 1.6). Third crossings were made just past halfway through the trial (*Mdn* =57.7%, *MAD* =6.85%), and fourth crossings (*Mdn* =69.4%, *MAD* =5.90%) were made around the same time as secondary crossings in the one-template condition, *BF*_10_ = 0.2, *d* = −0.1.

The number of unique templates fixated in the 4-Unlimited condition (Figure 4b) suggests two principal strategies: Some participants fixated (i.e., attempted to encode) approximately one template per crossing, in all crossings, thus loading VWM minimally. Other participants rather fixated multiple templates in their first crossing, and fewer templates in subsequent crossings. Most of the latter group of participants (who averaged three or more fixated templates in their initial crossing) still fixated approximately two unique templates in their secondary crossing, which suggests that these participants tried to rely more on memory, but were not always successful in that attempt.

In sum, these findings suggest that resampling was used in two primary ways; either to double-check whether the target indeed matched the template, or as a means to only partially encode (a subset of) the templates in the initial crossing. Subsequent crossings could then be used to just-in-time encode remaining templates, or to strengthen existing VWM representations, if necessary.

### Usefulness of template resampling

#### Analysis

Given that participants could use external sampling both to just-in-time encode subsets and to refresh existing representations in VWM, we investigated more specifically how these strategies were applied. 1) The Number of Gaze Crossings to the template area provides a measure of whether resampling was applied differently in target-absent versus target-present trials. In target-present trials, search could regularly terminate before all templates were encoded. Conversely, participants needed to compare all templates against the search array when there was no target. A greater number of crossings in target-absent than target-present trials would therefore be expected in exhaustive search. This variable was aggregated by the mean number of crossings per participant, per condition. 2) Resampling could also serve to refresh existing template representations in VWM. We computed Gaze Ended on Templates; the percentage of trials in which the last fixation of the trial occurred in the template area, meaning that a response was given while (or directly after) fixating a template. Although not comprehensive, this outcome variable represents the majority of instances in which participants double-checked template representations in VWM.

The percentage of trials in which the gaze ended on the templates in the 1-Unlimited condition was analysed by performing a one-sample *t* test against 0, because there were no such occurrences of double-checking in the incorrect trials.

#### Results

**Figure 5.**
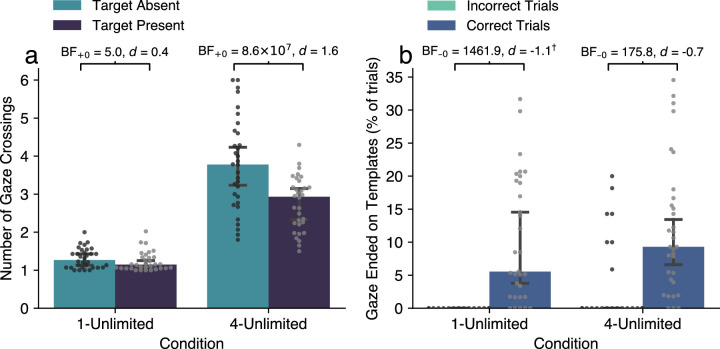
The ability to resample was used advantageously in both one- and four-template conditions, and in target-absent and target-present trials. (a) The number of gaze crossings per trial as an indicator of the degree of resampling. Split by condition and by target presence. (b) The percentage of trials in which the gaze ended in the template area, as an indicator of “double-checking” behavior. Split by condition and by incorrectly/correctly-answered trials. Note: Markers denote individual participants, aggregated over all trials. Bars display medians over participants with 95% CIs around the mean. † Results from a one-sample *t* test against 0.

Participants made consistently more gaze crossings to the template area in target-absent trials than in target-present trials (main effect *BF*_10_ = 29317.57, $\eta_{p}^{2}=0.77$), in both the 1-Unlimited condition (*BF*_+0_ = 5.0, *d* = 0.4) and in the 4-Unlimited condition (*BF*_+0_ = 8.57 × 10^7^, *d* = 1.6; Figure 5a). In the 4-Unlimited condition, participants crossed nearly four times per trial (*Mdn* = 3.77, *MAD* = 1.29) in target-absent trials, which suggests that they inspected the templates more exhaustively in those trials, and applied resampling dynamically to verify that there was indeed no target.

Furthermore, participants’ gaze ended in the template area (indicative of double-checking behavior) more frequently in correctly answered trials than in incorrect trials in both conditions (main effect of correctness *BF*_10_ = 429.84, $\eta_{p}^{2}=0.49$; Figure 5b), which suggests that double-checking was a useful strategy for achieving greater accuracy. There was no main effect of the number of templates (*BF*_10_ = 0.53, $\eta_{p}^{2}=0.07$), which indicates that participants used double-checking equally often in both one- and four-template conditions. Although the absolute percentages of correctly answered trials in which this behavior occurred were relatively low (1-Unlimited *Mdn* =5.6%, *MAD* =8.24%; 4-Unlimited *Mdn* =9.3%, *MAD* =8.93%), there was a clear link between double-checking at the end of trials and increased accuracy on the task.

In sum, participants dynamically used externally available templates to their advantage across conditions. For instance, they resampled more often to verify target absence, and used double-checking at the end of the trial to achieve greater accuracy.

## General discussion

The role of VWM in visual search has been studied almost exclusively with templates which can only be memorized *before* starting search (e.g., Olivers & Eimer, 2011; van Moorselaar, Theeuwes, & Olivers, 2014; Bahle, Beck, & Hollingworth, 2018). The external world, however, frequently provides possibilities to offload memory to the environment or to refresh template representations in memory during search. Across two experiments, we investigated whether participants delayed the encoding of templates when external templates remained available, whether they refreshed existing template representations, and how this ultimately affected task performance. Results showed that participants used external templates in all conditions that allowed it, in particular by delaying encoding (in line with predictions from VWM research; O’Regan, 1992; Ballard, Hayhoe, & Pelz, 1995; Droll et al., 2005; Risko & Dunn, 2015; Risko & Gilbert, 2016; Somai et al., 2020; Van der Stigchel, 2020; Draschkow et al., 2021), or by refreshing their existing internal template representations (conform e.g., Alfandari et al., 2019).

Does resampling aid visual search, and if so, in what way? A benefit of the possibility to resample (i.e., on accuracy and completion time; Palmer et al., 2000; Hulleman & Olivers, 2017; Wolfe, 2021) was present in all but the easiest condition (with one relatively simple template), and this benefit scaled as search became more difficult (complex stimuli and more templates; Bethell-Fox & Shepard, 1988; Eng, Chen, & Jiang, 2005; Drew & Wolfe, 2014; van Moorselaar et al., 2014; Ort & Olivers, 2020). First, participants spent less time dwelling on the templates in their initial inspection when they could resample, compared to when they could not. Second, participants made shorter fixations on individual templates when they could resample, which suggests that they attempted to encode the fixated templates less deeply and thereby relied less on internal storage in VWM. We argue that participants spent less time encoding templates when they could resample, because potentially insufficient representations could simply be refreshed later in the trial. This reliance on external templates, relative to fully loading VWM, was therefore temporally efficient in such a way that it offset the cost of making additional saccades between the search and template areas. As such, being able to resample templates allows for decreased VWM usage in terms of the number of encoded templates and depth of encoding, which in turn provides a clear time benefit to search.

Resampling also provided participants with ways to boost confidence during search, thereby increasing accuracy on the task. Specifically, participants resampled the template area more often in target-absent trials than in target-present trials as a way of verifying that indeed no target was present. Furthermore, participants occasionally refixated the template area directly before giving a response, thereby refreshing existing template representations in VWM, which was linked to higher accuracy. Together, these findings highlight that resampling can benefit accuracy in multiple ways.

Interestingly, the fact that participants could fixate templates for verification at the end of trials must mean that not only template representations were encoded in VWM, but that some elements of the search array were also in memory, not only as elements which help guide search (as in Wolfe, 2021), but also as target templates. In instances of search where external templates remain available, templates and targets can therefore serve interchangeable roles throughout search and within VWM (reminiscent of hybrid search; Drew, Boettcher, & Wolfe, 2017; Li, Chen, Wolfe, & Olivers, 2023).

Irrespective of strategy, almost all participants could perform the task at above-chance level (even at the highest difficulty), which suggests that resampling was generally not strictly necessary. However, there were individual differences regarding the number of templates that were fixated in the initial crossing; some participants encoded one template at a time, whereas other participants attempted to encode multiple templates in each crossing. These individual differences in strategies could in turn relate to individual differences in, for example, VWM capacity or executive functioning.

Furthermore, it is likely that not only the number of templates and stimulus set influence the degree of external (re)sampling. Varying other aspects of the task, such as the number of distractors, stimulus size, and crowding, may further modulate how frequently templates are resampled. Because distractors could here occur multiple times within the search array (thereby decreasing search difficulty; Duncan & Humphreys, 1989), the degree of resampling may have been higher if distractors could not occur redundantly.

In the framework of guided search (and alternative models), the final step consists of comparing an attended item in the search array to the template in memory (Palmer et al., 2000; Wolfe, 2021). Extending this framework, we suggest that not only the search array can be refixated, but that template representations in VWM may also be resampled before a decision is made. In many instances of search, the external world can therefore not only provide us with the challenge (find a target), but can also ease the challenge (by allowing us to refresh the template or to delay encoding).

## Conclusion

While visual search is commonly studied with to-be-memorized, and subsequently unavailable, search templates, many instances of search are clearly different. For instance, we might be desperate while trying to find that missing screw when assembling a new cupboard, but fortunately we can refresh the template representation by looking back at the manual. Participants frequently revisited templates during search when they were given the chance, and more so when search was difficult. How participants used external sampling hereby differed; in some instances participants encoded only subsets of templates, in other instances participants double-checked, both of which benefited search performance. Given that we can resample templates in many instances of visual search, which is often beneficial to task performance, we strongly advise not to hide your Swedish furniture assembly instruction manual. These findings bear implications for influential models of visual search, which should consider the option that not only the search array, but also external templates, can be resampled.

## Supplementary Material

Supplement 1

## References

[bib1] Adam, K. C., Vogel, E. K., & Awh, E. (2017). Clear evidence for item limits in visual working memory. *Cognitive Psychology,* 97, 79–97, doi:10.1016/j.cogpsych.2017.07.001. 28734172 PMC5565211

[bib2] Alfandari, D., Belopolsky, A. V., & Olivers, C. N. L. (2019). Eye movements reveal learning and information-seeking in attentional template acquisition. *Visual Cognition,* 27(5–8), 467–486, doi:10.1080/13506285.2019.1636918.

[bib3] Anderson, J. R. (1996). A simple theory of complex cognition. *American Psychologist,* 51(4), 355–365, doi:10.1037/0003-066X.51.4.355.

[bib4] Arnoult, M. D. (1956). Familiarity and recognition of nonsense shapes. *Journal of Experimental Psychology,* 51(4), 269–276, doi:10.1037/h0047772. 13306876

[bib5] Bahle, B., Beck, V. M., & Hollingworth, A. (2018). The architecture of interaction between visual working memory and visual attention. *Journal of Experimental Psychology: Human Perception and Performance,* 44(7), 992–1011, doi:10.1037/xhp0000509. 29629781 PMC6037540

[bib6] Ballard, D. H., Hayhoe, M. M., & Pelz, J. B. (1995). Memory representations in natural tasks. *Journal of Cognitive Neuroscience,* 7(1), 66–80, doi:10.1162/jocn.1995.7.1.66. 23961754

[bib7] Becker, S. I. (2011). Determinants of dwell time in visual search: Similarity or perceptual difficulty? *PLoS One,* 6(3), e17740, doi:10.1371/journal.pone.0017740. 21408139 PMC3050928

[bib8] Bethell-Fox, C. E., & Shepard, R. N. (1988). Mental rotation: Effects of stimulus complexity and familiarity. *Journal of Experimental Psychology: Human Perception and Performance,* 14, 12–23, doi:10.1037/0096-1523.14.1.12. 2964504

[bib9] Brodersen, K. H., Ong, C. S., Stephan, K. E., & Buhmann, J. M. (2010). The Balanced Accuracy and Its Posterior Distribution. In *20th International Conference on Pattern Recognition, August 2010, Istanbul, Turkey* (pp. 3121–3124).

[bib10] Cain, M. S., Adamo, S. H., & Mitroff, S. R. (2013). A taxonomy of errors in multiple-target visual search. *Visual Cognition,* 21(7), 899–921, doi:10.1080/13506285.2013.843627.

[bib11] Carlisle, N. B., Arita, J. T., Pardo, D., & Woodman, G. F. (2011). Attentional templates in visual working memory. *Journal of Neuroscience,* 31(25), 9315–9322, doi:10.1523/JNEUROSCI.1097-11.2011. 21697381 PMC3147306

[bib12] Dalmaijer, E. S., Mathôt, S., & Stigchel, S. Van der. (2014). PyGaze: An open-source, cross-platform toolbox for minimaleffort programming of eyetracking experiments. *Behavior Research Methods,* 46(4), 913–921, doi:10.3758/s13428-013-0422-2. 24258321

[bib13] Draschkow, D., Kallmayer, M., & Nobre, A. C. (2021). When natural behavior engages working memory. *Current Biology,* 31(4), 869–874.e5, doi:10.1016/j.cub.2020.11.013. 33278355 PMC7902904

[bib14] Drew, T., Boettcher, S. E., & Wolfe, J. M. (2017). One visual search, many memory searches: An eye-tracking investigation of hybrid search. *Journal of Vision,* 17(11), 1–10, doi:10.1167/17.11.5. PMC5596794 28892812

[bib15] Drew, T., & Wolfe, J. M. (2014). Hybrid search in the temporal domain: Evidence for rapid, serial logarithmic search through memory. *Attention, Perception & Psychophysics,* 76(2), 296–303, doi:10.3758/s13414-013-0606-y. PMC4350328 24343519

[bib16] Droll, J. A., & Hayhoe, M. M. (2007). Trade-offs between gaze and working memory use. *Journal of Experimental Psychology: Human Perception and Performance,* 33(6), 1352–1365, doi:10.1037/0096-1523.33.6.1352. 18085948

[bib17] Droll, J. A., Hayhoe, M. M., Triesch, J., & Sullivan, B. T. (2005). Task demands control acquisition and storage of visual information. *Journal of Experimental Psychology: Human Perception and Performance,* 31, 1416–1438, doi:10.1037/0096-1523.31.6.1416. 16366799

[bib18] Duncan, J., & Humphreys, G. W. (1989). Visual search and stimulus similarity. *Psychological Review,* 96, 433–458, doi:10.1037/0033-295X.96.3.433. 2756067

[bib19] Eng, H. Y., Chen, D., & Jiang, Y. (2005). Visual working memory for simple and complex visual stimuli. *Psychonomic Bulletin & Review,* 12(6), 1127–1133, doi:10.3758/BF03206454. 16615339

[bib20] Hansen, J. P., Mardanbegi, D., Biermann, F., & Bakgaard, P. (2018). A gaze interactive assembly instruction with pupillometric recording. *Behavior Research Methods,* 50(4), 1723–1733, doi:10.3758/s13428-018-1074-z. 29981049

[bib21] Hayhoe, M. M., Shrivastava, A., Mruczek, R., & Pelz, J. B. (2003). Visual memory and motor planning in a natural task. *Journal of Vision,* 3(1), 6, doi:10.1167/3.1.6. 12678625

[bib22] Irwin, D. E. (2004). Fixation location and fixation duration as indices of cognitive processing. *The Interface of Language, Vision, and Action: Eye Movements and the Visual World,* 217, 105–133.

[bib23] Hulleman, J., & Olivers, C. N. (2017). The impending demise of the item in visual search. *Behavioral and Brain Sciences,* 40, e132. 26673054 10.1017/S0140525X15002794

[bib24] JASP Team. (2022). JASP (Version 0.16.3)[Computer software]. Available from https://jasp-stats.org/.

[bib25] Kingsley, H. L. (1932). An experimental study of ‘search’. *American Journal of Psychology,* 44(2), 314–318.

[bib26] Koevoet, D., Naber, M., Strauch, C., Somai, R. S., & Stigchel, S. Van der. (2023). Differential aspects of attention predict the depth of visual working memory encoding: Evidence from pupillometry. *Journal of Vision,* 23(6), 9, doi:10.1167/jov.23.6.9. PMC10278550 37318440

[bib27] Li, A., Chen, Z., Wolfe, J. M., & Olivers, C. N. L. (2023). How do people find pairs? *Journal of Experimental Psychology General*, doi:10.1037/xge0001390. 36951742

[bib28] Luck, S. J., & Vogel, E. K. (2013). Visual working memory capacity: From psychophysics and neurobiology to individual differences. *Trends in Cognitive Sciences,* 17(8), 391–400. 23850263 10.1016/j.tics.2013.06.006 PMC3729738

[bib29] Melnik, A., Schüler, F., Rothkopf, C. A., & König, P. (2018). The world as an external memory: The price of saccades in a sensorimotor task. *Frontiers in Behavioral Neuroscience,* 12, 253. 30515084 10.3389/fnbeh.2018.00253 PMC6255858

[bib30] Moorselaar, D. van, Theeuwes, J., & Olivers, C. N. (2014). In competition for the attentional template: Can multiple items within visual working memory guide attention? *Journal of Experimental Psychology: Human Perception and Performance,* 40(4), 1450–1464, doi:10.1037/a0036229. 24730738

[bib31] Olivers, C. N., & Eimer, M. (2011). On the difference between working memory and attentional set. *Neuropsychologia,* 49(6), 1553–1558, doi:10.1016/jg.neuropsychologia.2010.11.033. 21145332

[bib32] O'Regan, J. K. (1992). Solving the “real” mysteries of visual perception: The world as an outside memory. *Canadian Journal of Psychology/Revue canadienne de psychologie,* 46(3), 461, doi:10.1037/h0084327. 1486554

[bib33] Ort, E., & Olivers, C. N. (2020). The capacity of multiple-target search. *Visual Cognition,* 28(5–8), 330–355, doi:10.1080/13506285.2020.1772430.

[bib34] Palmer, E. M., Van Wert, M. J., Horowitz, T. S., & Wolfe, J. M. (2019). Measuring the time course of selection during visual search. *Attention, Perception, & Psychophysics,* 81(1), 47–60, doi:10.3758/s13414-018-1596-6. 30242674

[bib35] Palmer, J., Verghese, P., & Pavel, M. (2000). The psychophysics of visual search. *Vision Research,* 40(10), 1227–1268, doi:10.1016/S0042-6989(99)00244-8. 10788638

[bib36] Pedregosa, F., Varoquaux, G., Gramfort, A., Michel, V., Thirion, B., Grisel, O., et al. (2011). Scikit-learn: Machine learning in Python. *Journal of Machine Learning Research,* 12, 2825–2830.

[bib37] Risko, E. F., & Dunn, T. L. (2015). Storing information in-the-world: Metacognition and cognitive offloading in a short-term memory task. *Consciousness and Cognition,* 36, 61–74, doi:10.1016/j.concog.2015.05.014. 26092219

[bib38] Risko, E. F., & Gilbert, S. J. (2016). Cognitive offloading. *Trends in Cognitive Sciences,* 20(9), 676–688, doi:10.1016/J.TICS.2016.07.002. 27542527

[bib39] Sahakian, A., Gayet, S., Paffen, C. L. E., & Stigchel, S. Van der. (2023). Mountains of memory in a sea of uncertainty: Sampling the external world despite useful information in visual working memory. *Cognition,* 234, 105381, doi:10.1016/j.cognition.2023.105381. 36724621

[bib40] Smith, N. D., Crabb, D. P., & Garway-Heath, D. F. (2011). An exploratory study of visual search performance in glaucoma: Study of visual search performance in glaucoma. *Ophthalmic and Physiological Optics,* 31(3), 225–232, doi:10.1111/j.1475-1313.2011.00836.x. 21470272

[bib41] Somai, R. S., Schut, M. J., & Stigchel, S. Van der. (2020). Evidence for the world as an external memory: A trade-off between internal and external visual memory storage. *Cortex,* 122, 108–114, doi:10.1016/j.cortex.2018.12.017. 30685062

[bib43] Sullivan, B., Ludwig, C. J. H., Damen, D., Mayol-Cuevas,W., & Gilchrist, I. D. (2021). Look-ahead fixations during visuomotor behavior: Evidence from assembling a camping tent. *Journal of Vision,* 21(3), 13, doi:10.1167/jov.21.3.13. PMC7961111 33688920

[bib44] Titchener, E. B. (1924). The overlooking of familiar objects. *American Journal of Psychology,* 35(2), 304–305, doi:10.2307/1413844.

[bib45] Triesch, J., Ballard, D. H., Hayhoe, M. M., & Sullivan, B. T. (2003). What you see is what you need. *Journal of Vision,* 3(1), 9, doi:10.1167/3.1.9. 12678628

[bib42] Van der Stigchel, S. (2020). An embodied account of visual working memory. *Visual Cognition,* 28(5–8), 414–419. 10.1080/13506285.2020.1818659 PMC8034609 33841024

[bib46] Vanyukov, P. M., Warren, T., Wheeler, M. E., & Reichle, E. D. (2012). The emergence of frequency effects in eye movements. *Cognition,* 123(1), 185–189, doi:10.1016/j.cognition.2011.12.011. 22264379 PMC3278573

[bib47] Vogel, E. K., & Awh, E. (2008). How to exploit diversity for scientific gain: Using individual differences to constrain cognitive theory. *Current Directions in Psychological Science,* 17(2), 171–176, doi:10.1111/j.1467-8721.2008.00569.x.

[bib48] Wolfe, J. M. (1994). Guided search 2.0 a revised model of visual search. *Psychonomic Bulletin & Review,* 1(2), 202–238, doi:10.3758/BF03200774. 24203471

[bib49] Wolfe, J. M. (1998). What can 1 million trials tell us about visual search? *Psychological Science,* 9(1), 33–39, doi:10.1111/1467-9280.00006.

[bib50] Wolfe, J. M. (2010). Visual search. *Current Biology,* 20(8), R346–R349. 21749949 10.1016/j.cub.2010.02.016 PMC5678963

[bib51] Wolfe, J. M. (2021). Guided search 6.0: An updated model of visual search. *Psychonomic Bulletin and Review,* 28(4), 1060–1092, doi:10.3758/s13423-020-01859-9. 33547630 PMC8965574

