# Supplementary material for: Don't hide the instruction manual: A dynamic trade-off between using internal and external templates during visual search
Source: J Vis. 2023 Jul 24;23(7):14. doi: 10.1167/jov.23.7.14 (PMC10382786; doi:10.1167/jov.23.7.14)
Supplement: Supplement 1 [file jovi-23-7-14_s001.pdf]

Supplementary Material to Don't hide the instruction manual: A dynamic trade-off between using internal and external templates during visual search

Alex J. Hoogerbrugge

Christoph Strauch

Tanja C. W. Nijboer

Stefan Van der Stigchel

Code and data availability

Experiment code and participant data are available via the Open Science Framework: https://osf.io/ec7b6/. Instructions are given in the README file. Analysis scripts, the resulting outcome variables, and JASP-files can be retrieved from the same sources. A reference file with all statistical outcomes can be retrieved at https://osf.io/qckf8. Animations of trials can be found in the animations folder.

Search duration

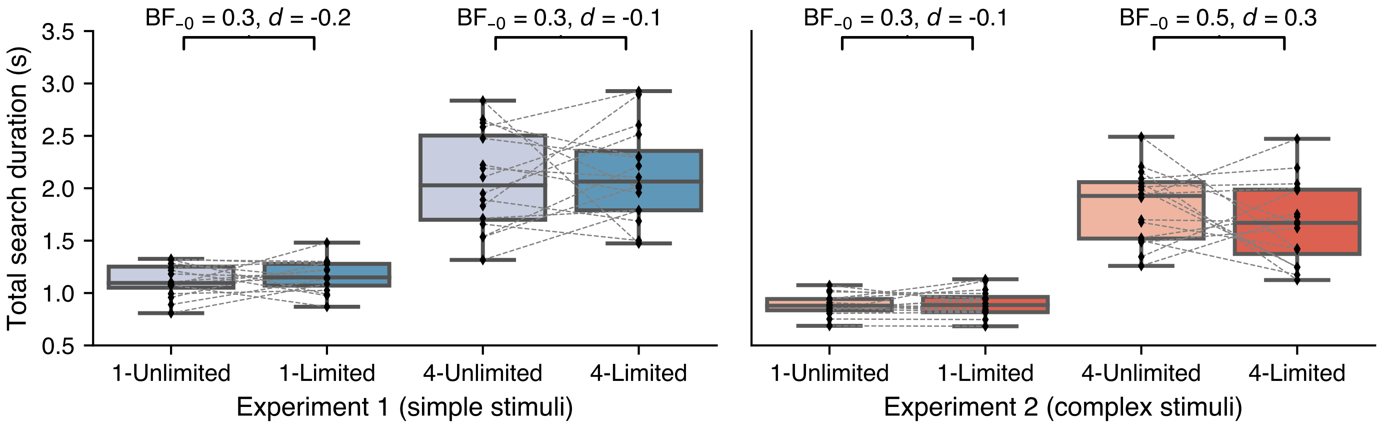

Figure 1: No differences in total search duration between Unlimited- and Limited conditions. Search duration was computed as the sum duration of all fixations in the search array.

Note: Both panels visualize data of correctly answered and matching trials only. Diamond markers denote individual participants.

No clear learning effects

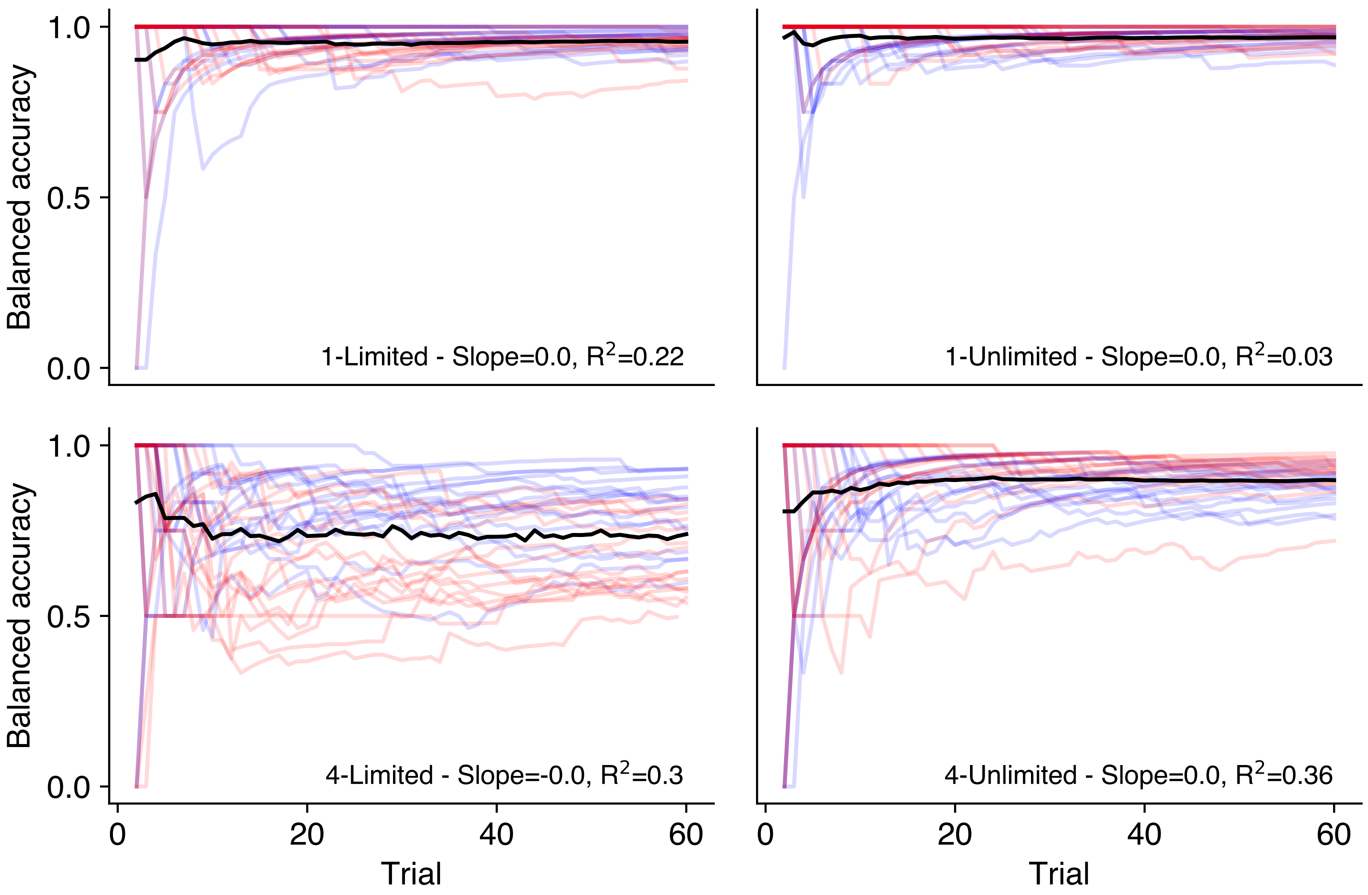

Figure 2: Balanced accuracy over trials. The accuracy of the first trial was always 0 or 1. Balanced accuracy converges and fluctuations become smaller as additional trials are taken into account. There was no clear learning effect (increase in accuracy) within blocks.

Note: Blue lines visualize participants in Experiment 1, red lines visualize participants in Experiment 2. The thick black line shows the median over all participants.

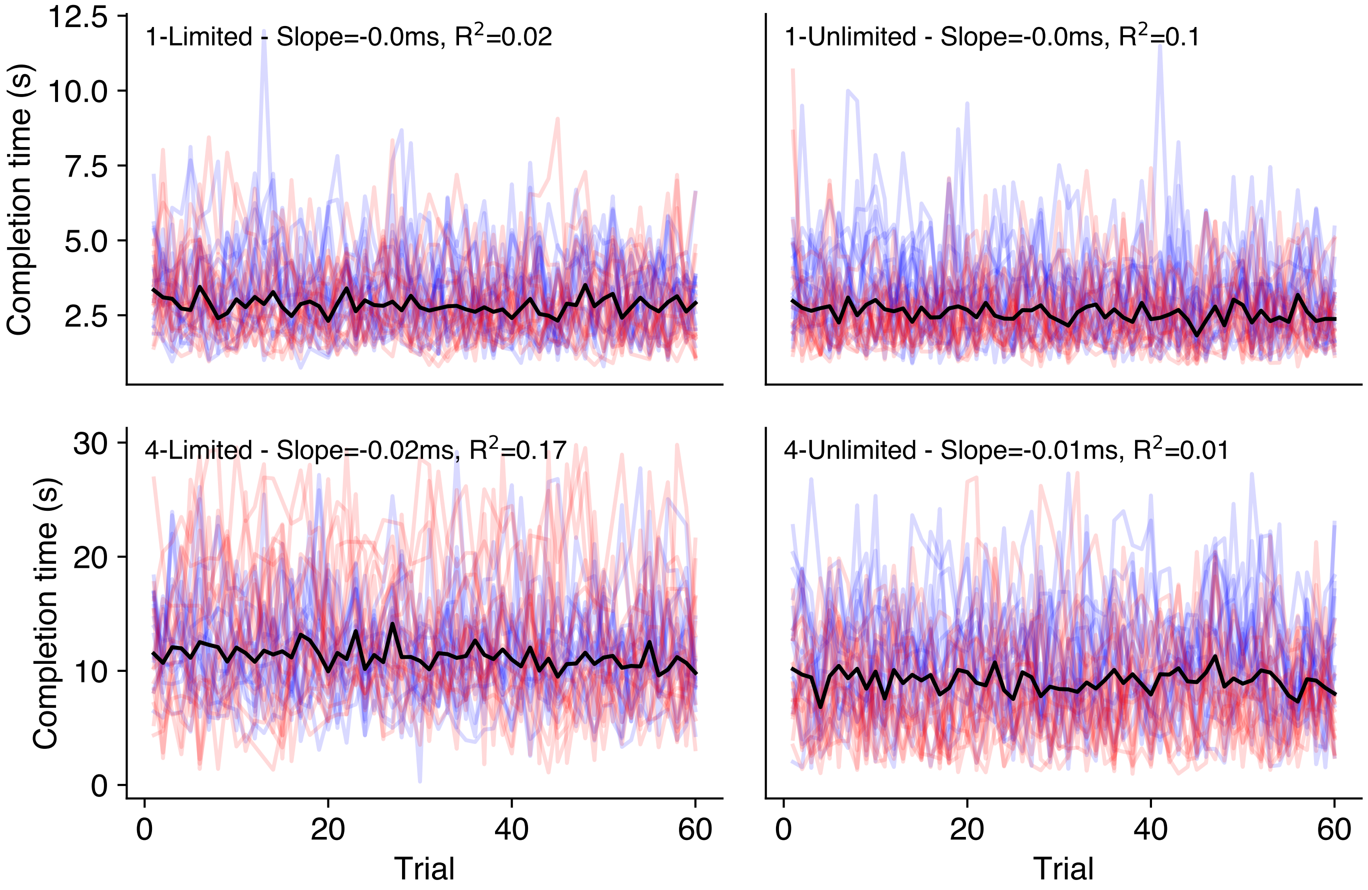

Figure 3: Completion time over trials. There was no clear learning effect (decrease in completion time) within blocks.

Note: Blue lines visualize participants in Experiment 1, red lines visualize participants in Experiment 2. The thick black line shows the median over all participants.
